# Discordance in Clinical Indicators With Sequential Fecal Microbiota Transplantation: A Case of Fulminant *Clostridioides Difficile* Infection

**DOI:** 10.14309/crj.0000000000001731

**Published:** 2025-06-11

**Authors:** Peter Bhandari, Reanay Berezovskiy, Salima Makhani, Valerie Gausman, Neelesh Rastogi, Sabina Braude

**Affiliations:** 1 Division of Gastroenterology, Morristown Medical Center, Atlantic Health System, Morristown, NJ; 2 New York Institute of Technology College of Osteopathic Medicine, Old Westbury, NY; 3 Division of Gastroenterology, Overlook Medical Center, Atlantic Health System, Summit, NJ

**Keywords:** *Fulminant clostridioides difficile*, *Clostridioides difficile colitis*, Pseudomembranous colitis, colonoscopy, fecal microbiota transplantation

## Abstract

Fulminant *Clostridioides difficile* infection (CDI) is a rare, severe type of CDI, often associated with extended hospitalizations, significant healthcare costs, and elevated mortality rates. Fecal microbiota transplantation remains an effective treatment modality for patients with fulminant CDI, with high cure rates reported after multiple treatments. Stool frequency, pseudomembrane resolution, and inflammatory markers are routinely monitored to evaluate disease severity and treatment responsiveness. Our case highlights a discordance in these indicators and demonstrates C-reactive protein as an important marker in assessing residual colitis and disease resolution. Comprehensive scoring systems should consider incorporating C-reactive protein and other biomarkers to optimize CDI management.

## INTRODUCTION

*Clostridioides difficile* infection (CDI) is a leading cause of hospital acquired infections and infectious diarrhea. Risk factors include prolonged hospitalization, advanced age, immunocompromised states, and most importantly the use of broad-spectrum antibiotics.^1^ Antibiotics disrupt normal gut flora, enabling *C. difficile* to proliferate and produce 3 toxins: toxin A, toxin B, and binary toxin. Toxins A and B cause cytotoxicity and intestinal epithelial damage, while binary toxin serves to amplify their effects.^2^ Diagnosis is primarily clinical and is confirmed through stool assays that detect *C. difficile* toxins or molecular testing.

Fulminant CDI (FCDI) is a severe, life-threatening form of the disease and occurs in approximately 3%–8% of all CDI cases. It is characterized by the development of hypotension, shock, toxic megacolon/ileus, or multiorgan failure.^3^ Fulminant CDI also poses significant economic challenges, often requiring prolonged hospital stays, intensive care, and surgical interventions.

Fecal microbiota transplantation (FMT) has emerged as a promising treatment that significantly reduces mortality and the need for colectomy, with cure rates reaching upward of 80% in patients with fulminant CDI.^4,5^ Typically, inflammatory markers such as C-reactive protein (CRP) and white blood cell (WBC) count correlate with disease severity and treatment responsiveness.^6,7^ However, our case highlights that this relationship is not always straightforward as there was a notable discordance observed between clinical findings and inflammatory markers.

## CASE REPORT

An 84-year-old man with hypertension, atrial fibrillation, and moderately differentiated sigmoid adenocarcinoma who recently underwent a laparoscopic low anterior resection with anastomosis presented to the hospital on postoperative day 7 with abdominal pain. On arrival, his vitals were normal. Physical examination revealed mild lower abdominal tenderness with no peritoneal signs. Initial laboratory investigations were significant for leukocytosis of 15,400 cells/μL and normocytic anemia of 9.1 g/dL. Initial computed tomography scan of the abdomen and pelvis demonstrated a highly attenuated collection with tract formation adjacent to the suture site, concerning for an anastomotic leak. He was started on intravenous piperacillin/tazobactam, however continued to have worsening leukocytosis with new-onset diarrhea, and was found to have CDI with a CRP of 104 mg/L and an Age, Treatment with systemic antibiotics, Leukocyte count, Albumin, Serum creatinine (ATLAS) score of 8. He was initially started on oral vancomycin 125 mg every 4 hours but transitioned to fidaxomicin 200 mg twice daily on day 4 of treatment due to limited clinical improvement and fidaxomicin's association with lower CDI recurrence rates and its narrower antibacterial spectrum, which helps preserve the gut microbiota.

**Figure 1.**
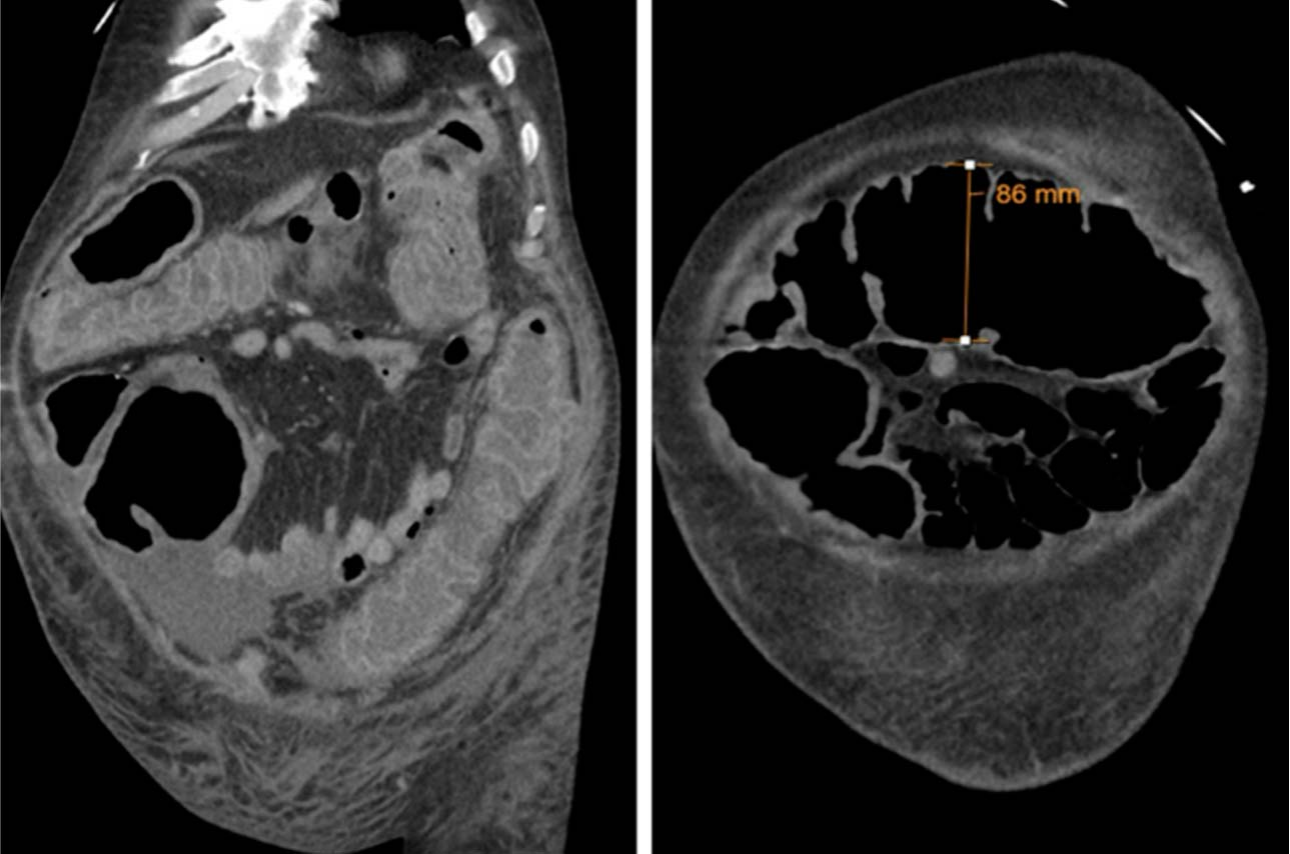
Interval computed tomography of the abdomen/pelvis with colonic dilation and circumferential wall thickening, consistent with toxic megacolon.

**Figure 2.**
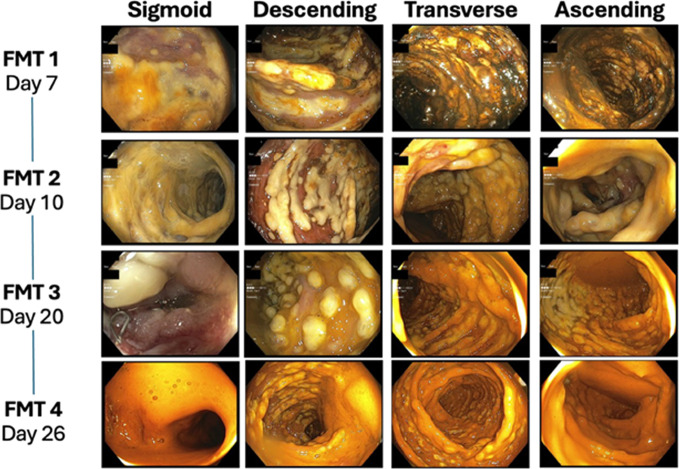
Endoscopic evaluation of the colon during sequential fecal microbiota transplantation treatments.

Interval computed tomography of the abdomen and pelvis revealed worsening colonic distention, measuring up to 8.6 cm in the transverse colon with circumferential wall thickening and no evidence of obstruction, concerning for megacolon (Figure 1). His antibiotic regimen was subsequently escalated to oral vancomycin 500 mg every 4 hours and intravenous metronidazole 500 mg every 8 hours as he had progressed to fulminant CDI. Owing to the patient's worsening condition and lack of response to standard antibiotic therapy, the decision was made to initiate FMT. He subsequently received 4 sequential FMTs for FCDI, all administered colonoscopically on days 7, 10, 20, and 26 with a discordance in inflammatory markers, clinical, and endoscopic findings (Figure 2 and Table 1). Stool microbiota analysis was not performed before or after FMTs. After the fourth FMT, he had significant clinical improvement and was discharged to a subacute rehab facility on oral fidaxomicin 200 mg twice daily. He declined further outpatient FMTs until completion of his rehabilitation program and tested negative for CDI 8 weeks later.

**Table 1.** Summary of FMT interventions, clinical status, and biomarker trends

Day of treatment	WBC (cells/µL)	CRP (mg/L)	Stool frequency	Colonoscopy findings	Clinical picture
Day 7 (FMT 1)	52.2 K (↑↑)	138 (↑↑)	5–6 (severe diarrhea)	Severe pseudomembranes, diffuse colonic involvement	Worsening colonic distention, no improvement with antibiotics
Day 10 (FMT 2)	60.1 K (↑↑)	37	3–4 (moderate diarrhea)	Persistent pseudomembranes, slight improvement	Some clinical improvement, but persistent colonic inflammation
Day 20 (FMT 3)	8.7 K (normalized)	152 (↑↑)	7–8 (worsening symptoms)	Moderate pseudomembranes, some mucosal healing	Persistent symptoms, discordant inflammatory markers, worsening stool frequency
Day 26 (FMT 4)	7.7 K (normalized)	88 (residual colitis)	2–4 (improved)	Marked resolution of pseudomembranes, mild residual inflammation	Significant clinical improvement, but residual colitis present
Discharge	Normalized	88 (persistent inflammation)	2–4 (stable)	Patient declined further treatment after discharge	Overall improvement, CDI negative, continued fidaxomicin

↑↑: Significantly increased risk of mortality and rapid disease progression.

CDI, *Clostridioides difficile* infection; CRP, C-reactive protein; FMT, fecal microbiota transplantation; WBC, white blood cell.

## DISCUSSION

Although fulminant CDI occurs infrequently, it is associated with mortality rates of 30%–40%.^8^ FMT remains an effective treatment for FCDI, with studies reporting cure rates of 81% after a single FMT and 92% after multiple treatments.^9^ An open-label trial using a pseudomembrane targeted protocol paired with 14 days of vancomycin achieved a 100% cure rate in a population in which 57% of cases were fulminant.^10^ FMT has also been shown to reduce mortality and the need for colectomy in fulminant and refractory CDI.^5^ The American College of Gastroenterology clinical guidelines suggest sequential FMTs every 3–5 days with continued oral vancomycin or fidaxomicin until pseudomembrane resolution. To facilitate the reconstitution of the gut microbiome, a 24–48-hour interval should be maintained between each FMT and the resumption of antibiotics.^11^ If pseudomembranes persist but significant clinical improvement is noted, antibiotics should be continued for at least 5 days, followed by a final outpatient FMT. Our patient underwent FMT on days 7, 10, 20, and 26 with delays in the third and fourth sessions at the patient's request due to procedure intolerance, which was a notable limitation. This likely contributed to interval worsening symptoms and inflammatory markers on the latter FMTs. In addition, since administration was based on clinical deterioration rather than a standard protocol, this case may raise concerns about generalizability. The patient declined outpatient FMT and preferred to complete his treatment course with oral fidaxomicin. He ultimately tested negative for CDI after 8 weeks.

The American College of Gastroenterology also recommends monitoring the clinical response to FMT by evaluating stool frequency and consistency, pseudomembrane resolution, CRP levels, and WBC counts.^11^ Elevated CRP, increased WBC counts, and advanced age are recognized as independent risk factors for mortality in patients with CDI.^6,7,12^ Specifically, a CRP >149 mg/L and WBC >13,300 cells/µL have been associated with a significantly higher risk of mortality.^12^ By the time of discharge, our patient's WBC count normalized with overall clinical improvement in his stool frequency, decreasing to 2–4 per day. However, CRP levels remained elevated at 88 mg/L, reflecting persistent residual colitis, which was confirmed by colonoscopy. This is not to suggest that FMT was ineffective; on the contrary, it was highly effective in reducing pseudomembranes and colonic distention. However, residual inflammation persisted, which may guide clinical decisions such as subsequent FMT sessions or surgical interventions to achieve complete disease resolution.^13^

The ATLAS score is a widely used tool for assessing CDI severity at diagnosis, that can be used to predict the likelihood of severe outcomes such as colectomy/ileostomy, sepsis, and mortality.^14^ Although it outperforms other models in predicting CDI outcomes, it does not include some key markers of disease severity, such as CRP.^10,14^ This case highlights the importance of early recognition and timely treatment of fulminant CDI, the efficacy of FMT as a minimally invasive therapy, and the significant role of CRP in assessing residual colitis and disease resolution. Future research should focus on developing more comprehensive scoring systems that incorporate CRP and other biomarkers to optimize CDI management.

## DISCLOSURES

Author contributions: P. Bhandari and R. Berezovskiy wrote the manuscript. S. Makhani, V. Gausman, N. Rastogi, and S. Braude revised the manuscript. P. Bhandari is the article guarantor.

Financial disclosure: None to report.

Informed consent was obtained for this case report.
